# A rare adverse event of atorvastatin inducing leukocytoclastic vasculitis with ANCA‐negative (Anti‐Neutrophil cytoplasmic antibody) case report and literature review

**DOI:** 10.1002/ccr3.7030

**Published:** 2023-03-02

**Authors:** Nehemias Guevara‐Rodriguez, Mailing Flores‐Chang, Akhila Chilakala, Jose Contreras, Paula Perdomo, Gandrabur Liliya

**Affiliations:** ^1^ Department of Medicine, Internal Medicine St. Barnabas Hospital Health System Bronx New York USA; ^2^ CUNY School of Medicine, Medical school in New York City New York New York USA; ^3^ Internal Medicine, Department of Rheumatology St. Barnabas Hospital Health System Bronx New York USA

**Keywords:** ANCA, atorvastatin, drug reaction, Leukocytoclastic vasculitis, treatment, vasculitis

## Abstract

Leukocytoclastic vasculitis is an entity associated with drugs, infections, cryoglobulinemia, and connective tissue diseases but can also be idiopathic, systemic, or organ localized. Moreover, LCV associated with drugs is a rare disorder. When it is present usually has an elevation of anti‐neutrophil cytoplasmic antibody, most likely anti‐myeloperoxidase, which can be helpful to orient the diagnosis. We are presenting a 55‐year‐old female with a past medical history of diabetes mellitus (DM) and hyperlipidemia (HLD) who presented with a painful and pruritic rash localized in the abdomen and lower extremities that started 1 week after initiated atorvastatin for management of hyperlipidemia. This is the first case ever reported of leukocytoclastic vasculitis ANCA negative associated with atorvastatin, to our best knowledge.

## Abstract 2

Describe a case of leukocytoclastic vasculitis secondary to atorvastatin with a literature review. Explain the possible mechanism of action of leukocytoclastic vasculitis related to drugs. Create awareness of the medical community's potential adverse effects of broadly used medications such as atorvastatin.

## 1 INTRODUCTION

Vasculitis, defined as the inflammation of blood vessels, can be due to primary or secondary causes. Primary vasculitis results from an isolated inflammatory attack upon the blood vessels, and secondary vasculitis is due to an underlying health condition, but the clinical presentation between them is similar.^1^ Such a presentation can include localized or systemic signs and symptoms such as purpura, petechiae, fever, malaise, arthralgias/arthritis, peripheral blood eosinophilia, and any other organ involvement.^2^ Once determining the type and cause behind the vasculitis, it is crucial to decide on the extent of the vasculitis. This means assessing the vasculitis location and size, which can help in confirming the diagnosis.^3^

Once the diagnosis is determined, we can move to management and treatment. These can vary based on the type, cause, and degree of vasculitis. In our case, vasculitis was secondary to atorvastatin, and the treatment consisted of stopping the drug and taking moderate‐dose steroids for a short period.

Drug‐induced vasculitis often leads to ANCA‐associated vasculitis, and the offending agents that have been implicated include hydralazine, PTU, montelukast, and others.

Drug‐induced vasculitis can appear similar to primary vasculitis, so it can be hard to distinguish; furthermore, no specific test can confirm the causative drugs. One method to orient the diagnosis is the increase in ANCA levels, which can be assessed with high titers of anti‐myeloperoxidase.^4^ This noticeable increase also disappears with the removal of the drugs.^2^

Although not as commonly noted in the literature, one offending agent is a statin, given the rarity of its occurrence or underdiagnosis. Statins are widely used for the treatment of dyslipidemia. However, the adverse effects are mainly myopathy and muscle weakness when reported.^5^

## 2 CASE NARRATIVE

A 55‐year‐old female with a past medical history of diabetes mellitus (DM) and hyperlipidemia (HLD) presented to the emergency department (ED) with a painful and pruritic rash localized in the abdomen and lower extremities that started 2 weeks prior. The patient was started on statin medication for managing HLD by her primary care doctor 1 week before the pruritic rash began. She denied smoking, alcohol, or drug use.

Vital signs were blood pressure 135/86 mmHg, heart rate 98 beats/min, respiratory rate 19 breaths/min, temperature 97.9 F, and oxygen saturation 98% on room air.

**FIGURE 1:**
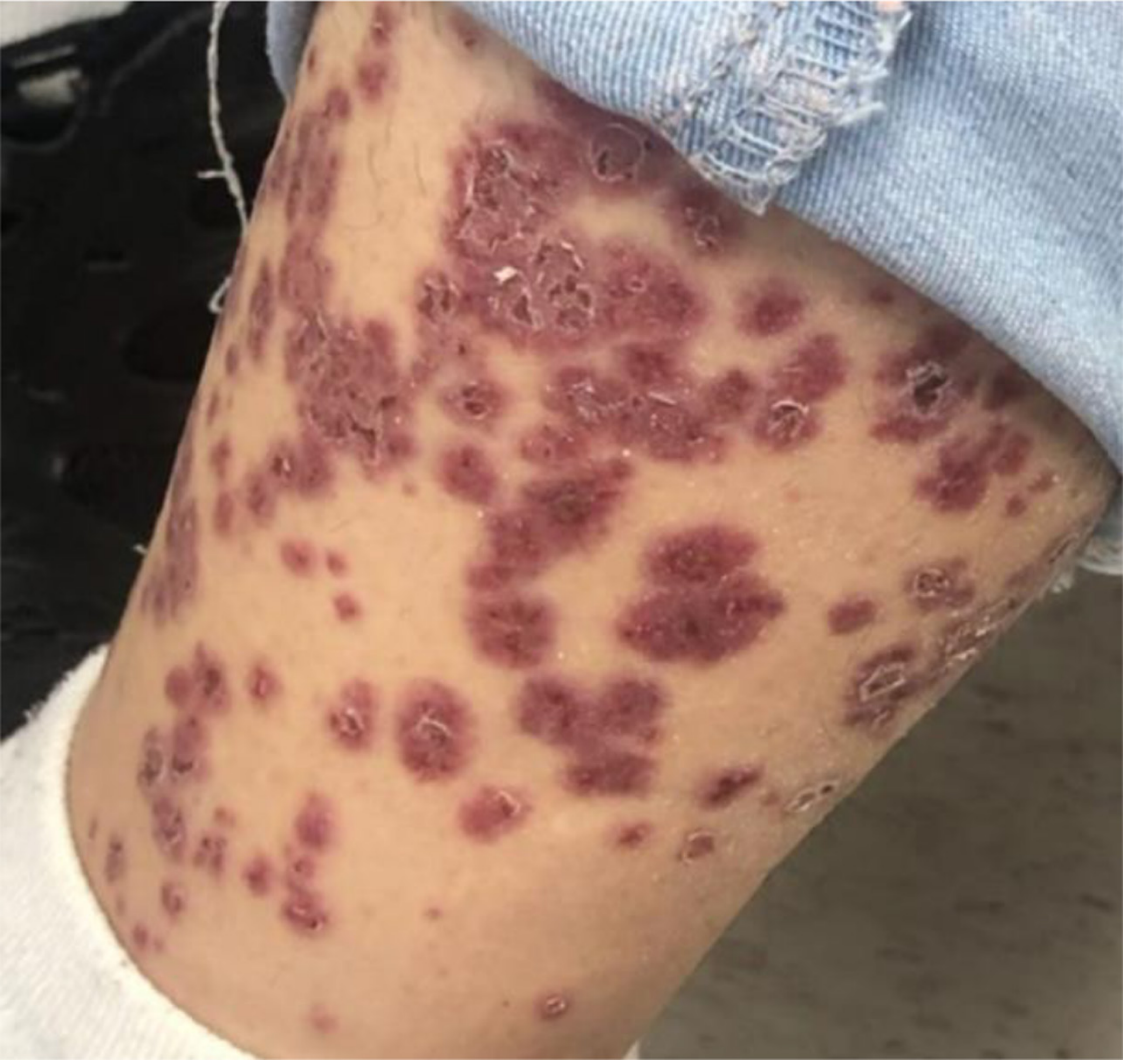
Multiple non‐blanching raised violaceous papules.

Physical examination was remarkable for multiple non‐blanching raised violaceous papules tender to palpation coalescing in certain areas in the lower extremities and less quantity in the lower abdomen. (Figure 1) Initial blood work that included ANCA with subtypes and hepatitis panel was unremarkable except for elevation of complement C3 (Table 1).

**TABLE 1:** Laboratory data.

Variable	On admission	Reference range
White cell count	7.2	4.2–9.1 10*3/uL
Neutrophils	49.6%	34.0%–67.9%
Lymphocytes	42.7%	21.8%–53.1%
Monocytes	6.6%	5.3%–12.2%
Eosinophils	0.1%	0.8%–7.0%
Hemoglobin	14.4	13.7%–17.5 gm./dL
Hematocrit	47.5	40.1%–51.0%
Platelet count	344	150–450 10*3/uL
MCV	81.2	79.0–92.2 fL
MCH	24.6	25.7–32.2 pg
MCHC	30.3	32.3–36.5 gm/dL
Sodium	136	135–145 mEq/L
Potassium	4.0	3.5–5.3 mEq/L
Chloride	99	96–108 mEq/L
Glucose	92	70–99 mg/dL
Calcium	9.4	9.2–11.0 mg/dL
Creatinine	0.8	0.6–1.2 mg/dL
ALT	21	4–36 IU/L
AST	24	8–33 IU/L
Bilirubin Total	0.6	0.1–1.2 mg/dL
Magnesium	1.7	1.3–2.1 mE Respond to Reviewer‐ Case of LCV associated with Atorvastatin. q/L
Complement C3	238	82–167 mg/dL
Complement C4	33	12–38 mg/dL
Antinuclear antibodies	Negative	Negative
HIV AG/AB combo test	Non‐reactive	
RPR	Non‐reactive	
Rheumatoid factor	Negative	
ESR	96	0–30 mm/hr
C‐Reactive Protein	3.68	0.00–1.0 mg/dL
Hepatitis B surface Ab	Reactive – 33.05	0.00–12.00 m[IU]/mL
Hepatitis B surface Ag	Non‐reactive	
Hepatitis B core Ab	Non‐reactive	
Hepatitis C surface Ag	Non‐reactive	
Serum immunoglobulins		
IgA	90	70–400 mg/dL
IgG	1000	700–1600 mg/dL
IgM	800	700–1600 mg/dL
Serum electrophoresis		
Protein total	7.0	6.0–8.5 g/dL
Albumin	4.0	2.9–4.4 g/dL
Alpha‐1 globulin	0.2	0.0–0.4 g/dL
Alpha‐2 globulin	0.7	0.4–1.0 g/dL
Beta globulin	0.8	0.7–1.3 g/dL
Gamma globulin	1.3	0.4–1.8 g/dL
M‐spike	Not observed	
A/G Ratio	1.3	0.7–1.7
ANCA Panel		
Cytoplasmic (C‐ANCA)	<1:20	Negative: <1:20 titer
Perinuclear (P‐ANCA)	<1:20	Negative: <1:20 titer
Atypical PANCA	<1:20	Negative: <1:20 titer
Antimyeloperoxidase abs (MPO)	<9.0	0.0–9.0 U/mL
Antiproteinase 3 abs (PR‐3)	<3.5	0.0–3.5 U/mL

**FIGURE 2:**
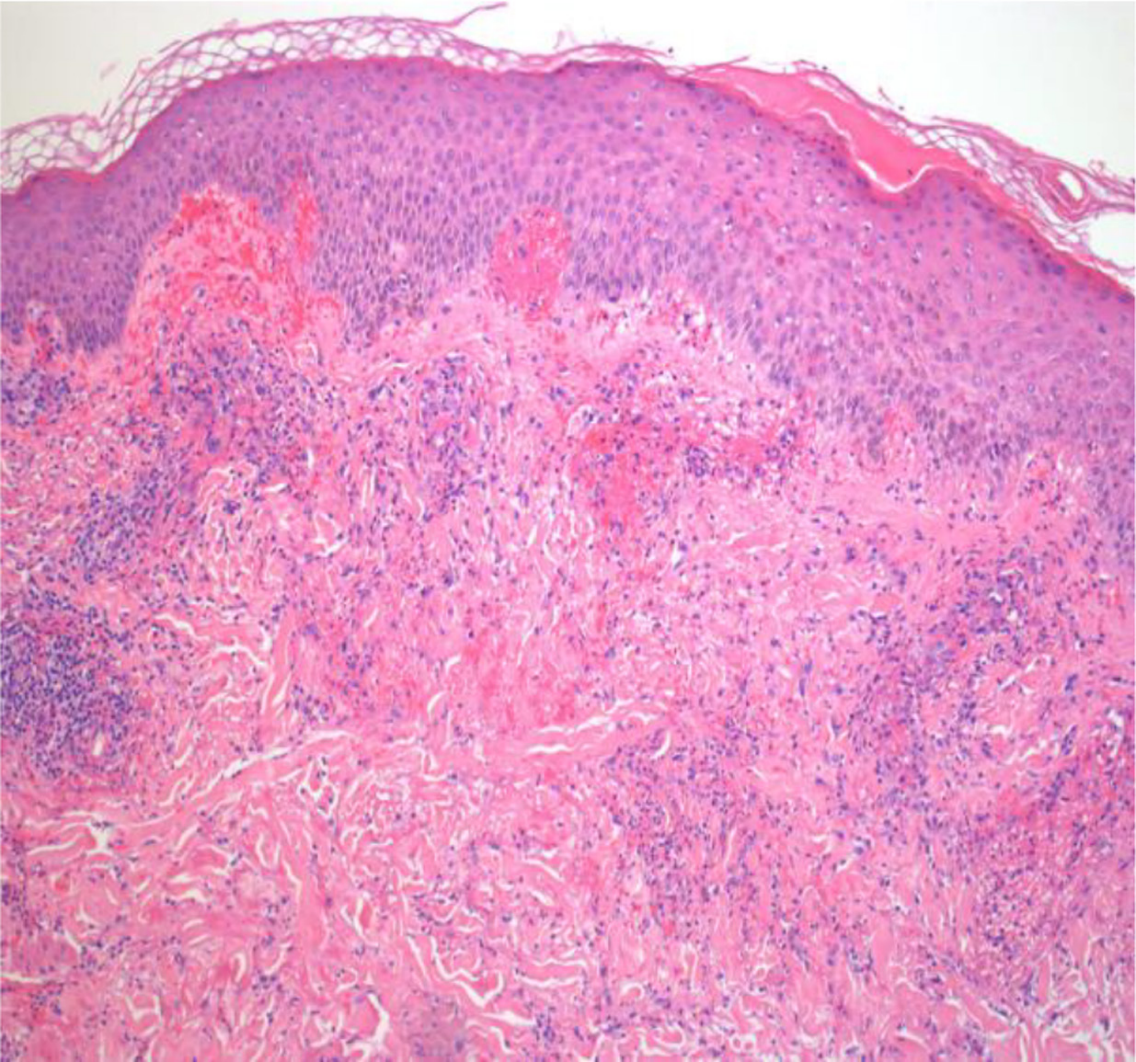
*Punch biopsy ‐ leukocytoclastic vasculitis*, sections show abundant mixed dermal inflammatory infiltrate composed of neutrophils and lymphocytes surrounding the walls of dermal and subcutaneous vessels. Blood vessels show moderate fibrinoid necrosis of walls and a background of red blood cell extravasation.

The patient underwent a punch biopsy that reported leukocytoclastic vasculitis [Figure 2]; immunofluorescence showed no deposition. Statin was immediately discontinued, and she was started on a 5‐day course of PO prednisone and a 2‐week course of antihistaminics with famotidine, hydroxyzine, along with triamcinolone ointment with a complete resolution of the lesion within the next 15 days. Diagnosis of leukocytoclastic vasculitis in the setting of drug use (statin) was made.

The patient is currently following up in an outpatient clinic with rheumatology and dermatology without recurrence of the disease or systemic signs/symptoms.

## 3 DISCUSSION

Statins are, without a doubt, a cornerstone of the treatment and prevention of cardiovascular disease; they are inhibitors of the conversion of 3‐hydroxy‐3‐methylglutaryl‐CoA (HMG‐CoA) into L‐mevalonate, which is the rate‐limiting step in cholesterol biosynthesis, this is achieved by competitive blocking of the active site of the enzyme HMG‐CoA reductase. Therefore, the blockade of the mevalonate pathway affects cholesterol production and ultimately reduces serum low‐density lipoprotein (LDL) cholesterol levels.

Statins have many other uses thanks to their anti‐inflammatory and immunomodulatory effects, pleiotropic effects (improvement of cardiovascular function, anti‐fibrotic effects, broad antioxidant, and anti‐inflammatory effects, enhancement of bone formation, and neuro‐ and renal‐protective effects),^6^, ^7^, ^8^, ^9^, ^10^, ^11^ reasons of which have been used in autoimmune disorders^12^, ^13^ and cancer diseases.^14^

However, statins are not exempt from severe adverse events; cases have been reported of rhabdomyolysis,^15^ lupus‐like syndrome,^16^ autoimmune diseases,^17^ neuromuscular diseases, pancreatitis, and hepatitis,^18^; however, even though the list is vast, the benefits overweight the adverse events.^19^, ^20^

Crestor has been associated with skin reactions <0.01%,^19^ to our best knowledge, and after extensive literature review, this is the first case of leukocytoclastic vasculitis induced by Atorvastatin ANCA negative.

Leukocytoclastic vasculitis is an entity that has been associated with drugs, infections, cryoglobulinemia, and connective tissue diseases but can also be idiopathic.^21^, ^22^

Symptoms can be organ localized or systemic; systemic involvement varies from 20 to 50% and is related to the triggered disease.^22^, ^23^, ^24^, ^25^, ^26^

Furthermore, relapse is associated with triggered‐based disease.  A study with a follow‐up of 3 years after the first episode demonstrated that the risk factors of chronic disease were cryoglobulins, arthralgia, normal temperature at diagnosis^21^, presence of ANCA‐positive, older age, persistent rash, abdominal pain, hematuria, the severity of the leukocytoclastic, and the absence of IgM deposit on the vessel walls^26^; it has been demonstrated as well by Alalwani et al. that the deposits of IgA are associated with a worse course of the disease (Gastrointestinal and renal involvement) and relapse of the same as well.^21^, ^22^, ^23^, ^24^, ^25^, ^26^, ^27^

However, LCV associated with drugs is not common.^22^, ^23^, ^24^, ^25^, ^26^, ^27^, ^28^ Even though some cases of vasculitis have been reported in the literature associated with statins, only one has been written using atorvastatin, which was associated with ANCA positives; however, the mechanism of vasculitis associated with statins (any of them) remains unknown.

Prasad T et al. reported 2 cases, and Haroon et al. reported 1 case of systemic vasculitis secondary to atorvastatin; all of them were ANCA positive, with unknown mechanisms; and all the cases presented complete resolution after stopping atorvastatin and treatment with steroids.^29^, ^30^, ^31^

The Food and Drug Administration (FDA) reported 54 cases of leukocytoclastic vasculitis associated with atorvastatin as part of the surveillance. Still, no proper case was reported, neither clinical history nor the clinical setting of the presentation.

Our patient did not present any antibody elevations. All the immunologic panel was negative, which makes a unique case. Based on the timeline of the clinical history, this LCV was secondary to atorvastatin.

Furthermore, the WHO‐UMC Naranjo score was 6 points which makes it probable; additionally, the patient just started the atorvastatin 2 months before and no other drug was given that could have triggered leukocytoclastic vasculitis.

The patient had a complete resolution of the vasculitis after stopping atorvastatin and taking a short course of steroids. The patient has been following up in our outpatient clinic without any systemic or localized manifestations of the disease.

## 4 CONCLUSION

Statins are a cornerstone in preventing and managing cardiovascular diseases and many diseases, given their effects beyond lowering lipids; however, they are not exempt from serious adverse events, such as LCV. The medical community should be aware of this association and its management.

## AUTHOR CONTRIBUTIONS

**Nehemias Antonio Guevara:** Conceptualization; formal analysis; project administration; supervision; validation; writing – original draft; writing – review and editing. **Mailing Flores:** Conceptualization; formal analysis; resources; writing – original draft; writing – review and editing. **Akhila Chilakala:** Writing – original draft. **Jose Contreras:** Resources; writing – original draft. **Paula Perdomo:** Resources; writing – original draft. **Liliya Gandrabur:** Conceptualization; data curation; formal analysis; methodology; supervision; validation; writing – review and editing.

## FUNDING INFORMATION

The authors received no financial support for this study.

## CONFLICT OF INTEREST STATEMENT

The authors report no conflicts of interest.

## CONSENT

Written informed consent was obtained from the patient to publish this report in accordance with the journal's patient consent policy.

## Data Availability

Data available on request due to privacy/ethical restrictions.
